# Mechanisms of FAI cartilage damage: experimental & simulation studies

**DOI:** 10.1186/1471-2474-16-S1-S5

**Published:** 2015-12-01

**Authors:** A Ng, E C Pegg, S J Mellon, D J Beard, D W Murray, H S Gill

**Affiliations:** 1 Nuffield Department of Orthopaedics, Rheumatology and Musculoskeletal Sciences, University of Oxford, Oxford, UK; 2 Centre for Regenerative Medicine, Department of Mechanical Engineering, University of Bath, Bath, UK

## 

Femoral acetabular impingement (FAI) is thought to be a key underlying reason for the development of osteoarthritis of the hip. There are two main types of FAI, cam-type and pincer-type. The cam-type FAI gives rise to cartilage delamination initially thought to occur on the acetabular side of the joint. The purpose of the current study was to look at the effects of cam-type impingement on the generation of shear strains at the bone/cartilage interface, using both experimental and finite element simulation methods. Sagittal slices (n=9) of femoral porcine cartilage-bone, 10 mm thick, were loaded using a five-axis custom test machine with a curved (radius 90 mm) steel indenter. The five-axis test machine allowed the samples to be subjected to compression and mixed compression/shear loading regimens. The specimen strains were measured using two dimensional digital image correlation (DIC). Each test was also simulated using finite element analysis, and the results compared with the DIC data. The specimens were then cyclically loaded either with or without damage to the cartilage layers; damage simulated clinically reported lesions. Maximal shear strain was found at the cartilage-bone interface, and was a function of compressive loading level. The finite element predictions matched the DIC measurements. The two parameters that were most important in terms of shear strain were the cartilage thickness and contact area radius. It was found that increased cartilage thickness and increased contact radius gave rise to higher shear strains. Cyclically loading the damaged specimens produced features of cartilage delamination consistent with clinical observations. The results of this study indicate high shear strain at the bone/cartilage interface is a possible mechanism leading to cartilage delamination, and may be the mechanism behind cartilage degradation in patients with cam-type FAI.

